# HIV-Encoded Gene Therapy as Anti-cancer Therapeutics: A Narrative Review

**DOI:** 10.7759/cureus.53431

**Published:** 2024-02-01

**Authors:** Pachamuthu Balakrishnan, Sankar Sathish, Shanmugam Saravanan

**Affiliations:** 1 Department of Microbiology, Saveetha Dental College and Hospitals, Saveetha Institute of Medical and Technical Sciences, Saveetha University, Chennai, IND

**Keywords:** viral vector, oral cavity squamous cell carcinoma, head and neck neoplasms, hiv aids, oncolytic virus therapy

## Abstract

Recently, there has been interest in using viruses as cancer treatments. Oncolytic virology was founded by scientists who noticed that viruses might preferentially lyse cancer cells over healthy ones. Oncolytic virotherapy has similar obstacles as other treatment approaches, gaining entry into the specific tumour cell, encountering antiviral immune responses, off-target infection and many other unfavourable circumstances in the tumour microenvironment, and a lack of unique therapeutic and predictive biomarkers. However, oncolytic viruses have emerged as the main players in the biological treatment for cancer with the use of vectors such as human adenoviruses in oncolytic virotherapy. Recent large-scale research has shown that other viruses, such as the measles virus and the herpes simplex virus (HSV), may potentially be viable options for cancer treatment. The FDA has cleared T-VEC, an HSV-based oncolytic virus, for use in biological cancer treatment after its successful completion of human clinical trials. Furthermore, the measles virus vaccine strain has shown remarkable outcomes in pre-clinical and clinical testing. The use of such modified viruses in biological cancer treatment holds promise for groundbreaking discoveries in the field of cancer research because of their therapeutic effectiveness, fewer side effects, and safety. Several other newer approaches have been used in recent years. HIV-encoded proteins are also hypothesized to promote mitochondrial homeostasis causing bystander-induced apoptosis. We provide an overview of the most recent developments in the clinical use of oncolytic virus-based biological cancer treatment in this study. This evaluation also assesses the advantages and disadvantages of the viral candidates and provides insight into their potential in the future.

## Introduction and background

Oral squamous cell carcinoma (OSCC) of the oral cavity represents one of the most common malignancies afflicting the head and neck worldwide. As per the data available from the Global Cancer Observatory (GCO), the annual incidence of OSCC was 377,713 cases worldwide, and also the highest number (>65%) was recorded in Asia in 2020 [1]. Historically, the treatment of OSCC has relied on conventional modalities that include surgical procedures, radiation therapy, chemotherapy, or a combination of all of these [2]. Several virus-mediated treatment strategies for OSCC are currently under investigation, and they are either in the pre-clinical and/or one of the other phases of clinical trials [3].

Oncolytic viruses (OVs) are multi-mechanistic therapeutic agents that can be genetically modified to multiply selectively in and destroy malignant tissues while sparing the normal host cells and at the same time restoring anti-malignant immunity, therefore offering a novel immune-therapeutic approach for the treatment of malignancy. In general, this anti-tumour effect of OVs acts in two ways, viz., by directly infecting and destroying tumour cells and/or by making the immune system work more stridently against tumour cells. Although both wild (non-engineered) and genetically modified (engineered) OVs have been shown to be inherently selective for malignant cells [4], the engineered OVs appear to possess an edge over the former in terms of safety and anti-tumour potential [5].

OVs are now being studied in clinical and experimental studies, and some clinical research has shown that OVs have a high safety profile and considerably improve the prognosis of cancer patients [6]. A clinical trial conducted with genetically modified OVs has revealed that the efficacy of oncolysis is synergistically enhanced while combining oncolytic virotherapy with other traditional anti-cancer therapies, such as chemotherapy and radiotherapy [7]. This review focuses on the current status of oncolytic virotherapy (OVT) and future potential options. 

## Review

OVT

The OVT approach works through viruses that are engineered to multiply in cancer cells and kill them while sparing healthy cells. One of the major benefits of OVTs over other cancer treatments is their ability to effectively amplify OVs via viral replication in tumour cells. OVs have been genetically modified to further boost their anti-tumour specificity, immunogenicity, and effectiveness in order to improve their usefulness as anti-cancer therapeutics [5,8]. The OV elicits anti-tumour immunity as a result of enhanced antigen cross-priming and immune cell recruitment into the tumour microenvironment (TME), which would effectively attack tumour cells. Secondly, an OV directly infects cancer cells, leading to oncolysis of these cell types in the TME. In 2005, the China FDA Department authorized H101 (Oncorine), the first recombinant oncolytic adenovirus, to be administered in conjunction with chemotherapy for the treatment of nasopharyngeal cancer. In 2015, the United States FDA (US-FDA) approved T-VEC (talimogene laherparepvec), the very first OV that displayed promising treatment for head and neck squamous cell carcinoma and melanoma. OVT has garnered significant attention because of the efficaciousness of OVs in human clinical studies. Following the success, several other viruses have been assessed for their activity, including poxvirus, reovirus, parvovirus, picornavirus, and the Newcastle disease virus (NDV) for its activity [2,7].

In T-VEC, the herpes simplex virus type 1 (HSV-1) was modified with the deletion of ICP47 and ICP34.5 genes and the insertion of the human granulocyte-macrophage colony-stimulating factor (GM-CSF) gene. T-VEC is a herpes simplex virus type 1 (HSV-1), in which the human granulocyte-macrophage colony-stimulating factor (GM-CSF) gene was inserted, and the infected cell protein (ICP)34.5 and ICP47 genes were deleted. The insertion of GM-CSF increases immune recognition by provoking anti-cancer immunity, and the deletion of the ICP34.5 and ICP47 genes improves safety, allows for tumour-specific replication, and provides immune evasion functions. Through a variety of mechanisms, OVs can lyse cancer cells and release pro-inflammatory cytokines, chemokines, and other hazard signals that aid in the recruitment and activation of immune cells within the TME. This results in the immune system recognizing the cancer and provoking anti-cancer immunity [9].

Enhancing the efficacy of OVT

**Figure 1:**
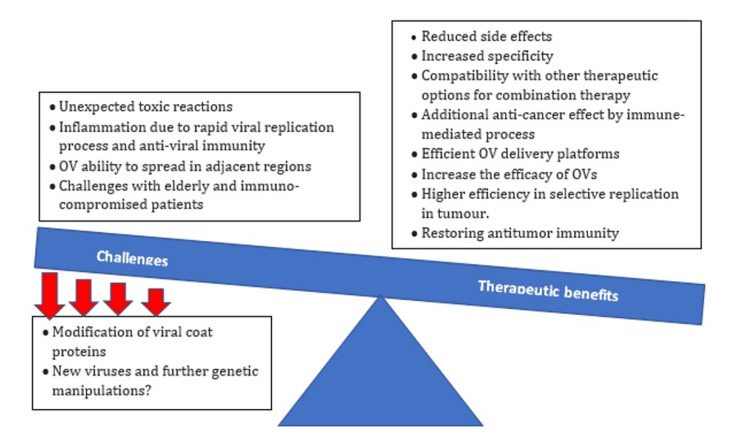
Challenges and benefits associated with the genetically modified viruses used in oncolytic viral therapy. Image Credit: Author Pachamuthu Balakrishnan

Notwithstanding that clinical trials, particularly with OV monotherapy, have not yielded robust antitumor efficacy (Figure 1), newer approaches hold the key to promising better therapeutic outcomes.

OVs are multi-functional cancer-fighters engineered to employ many diverse stratagems, and one of the strategies that we propose employs the DNA damage signal transduction cascade. Through this approach, the target HIV gene responsible for inflicting critical DNA damage in the host could be prudently used [8]. This would substantially damage the mitochondrial DNA causing the infected cancer cells to die via apoptosis. Strong evidence indicates that two Jurkat-derived cell lines containing silent HIV-1 provirus increased double-strand DNA breaks (DSBs) and phosphorylated histone H2AX on Ser139, a biomarker of DSBs. In addition, higher levels of phosphorylation of ATM at Ser1981, Chk2 at Thr68, and p53 at Ser15, which control the different components of the signalling pathways associated with DSBs are upregulated in the cells [9,10]. However, it has been documented that HIV-infected individuals who are antiretroviral therapy (ART)-naïve also experience mitochondrial DNA dysfunction and this has been documented in the Indian population by our group [6,10-13]. Differential modulation of mitochondrial activity is induced by HIV infection in different cell types. For example, among HIV-positive, ART-naïve patients, there is a positive correlation between the number of CD4+ T-cells and the change in mitochondrial membrane potential (ΔΨm).

One of the processes through which virally encoded proteins interact is mitochondrial dysregulation. HIV-induced cell death is linked to the virally encoded proteins env, vpr, tat, nef and vpu through mitochondrial activity. HIV-encoded proteins are hypothesized to promote mitochondrial homeostasis causing bystander-induced apoptosis [9,10]. However, more data need to be generated to prove this hypothesis. Therefore, this newer approach of arming engineered OVs with additional weaponry from the HIV gene that encodes the proteins could be potentially used after appropriate pre-clinical and clinical evaluations. 

Vector-assisted infection

For OVT to be more effective, it must efficiently overcome the factors that significantly reduce the therapeutic efficacy. The therapeutic virus is expected to initiate a robust early infection followed by rapid spread for enhanced efficacy [6]. Recombinant baculoviruses carrying immunodominant epitopes elicit strong immune responses in mice [6,14]. As baculoviruses can be made to express proteins in insect cells in large amounts, they have been utilized extensively for more than three decades as a method for gene expression [15]. Recombinant baculoviruses have demonstrated the ability to infiltrate diverse mammalian cells and express foreign genes utilizing mammalian promoters [16,17]. Baculoviruses can act as a successful gene expression vector for the baculovirus-induced immune response in mammalian cells. In suicide gene therapy, a toxin-coding gene and an enzyme-coding gene are introduced to target cancer cells to make them more sensitive to chemotherapy [17]. This approach has failed in the phase 3 clinical trial.

Adenoviruses are the most often utilized vector for tumour gene therapy. In order to express foreign antigens, they are also used in gene therapy and vaccinations. Certain important viral genes are removed from adenovirus vectors and substituted with a cassette expressing a foreign therapeutic gene, resulting in replication defects [16]. These vectors are used in cancer treatment, vaccine development, and gene therapy. Oncolytic, or replication-competent, vectors are used in cancer gene therapy. With the help of lytic viral replication, oncolytic vectors are made to propagate and destroy cancer cells only. Replication-defective and replication-competent adenovirus vectors are safe and effective therapeutic agents, according to several clinical investigations [18]. Replication-deficient adenoviruses and other recombinant adenoviruses such as conditionally replicating adenoviruses are now being widely studied for their potential use in cancer therapy. These conditionally replicating adenoviruses are reported to target specific cancer cells. Adenoviruses-based OVs are in the limelight and it is hoped that they constitute a successful gene therapy in cancers. The use of non-virulent viruses such as Reolysin, a wild-type reovirus variant with minimal virulence in normal human cells has been reported that displays oncolytic capabilities in cells with active Ras signalling. Reolysin (pelareorep) is being studied for targeting head and neck cancer. However, an engineered viral genome is considered to be a vital option for replication in the specifically targeted tumour cell because they are more effective in establishing stringent control over viral reproduction [6,16,18].

The vaccinia virus JX-594 (pexastimogene devacirepvec), which is used in a similar oncolytic viral treatment for hepatocellular carcinoma, is similarly close to receiving FDA clearance in North America and Europe. OVs including GM-CSF-expressing adenovirus CG0070 and HSV-1 virus G47∆, are being studied for the treatment of bladder carcinoma and glioblastoma. The NDV has a significant affinity for tumour cells and reproduces in the cytoplasm, breaking down the host genome or engaging in recombination. As one of the most promising oncolytic viruses employed in oncolytic treatment, it is safer and more appealing than retroviruses or certain other DNA viruses. Using the NDV as a gene delivery vector, reverse genetics technology was used to create recombinant NDV (rNDV), which has made NDV available for use in gene therapy techniques for the treatment of cancer and other disorders. Not only does rNDV provide stable expression of foreign therapeutic genes, but it can also boost the virus's capacity to eradicate tumour cells and trigger the host's immune system against tumours [19]. 

Immunostimulatory gene therapy started in the last two decades has now promising effects. Immunostimulatory gene therapy aims to enhance anti-tumour immunity by changing the course of the suppressive TME. Therefore, in order to fight the tumour from inside, the gene vehicle enters with an arsenal of weapons. The immune stimulators that activate and maintain Th1 responses are the most promising ones; yet, despite their dramatic effects in preclinical models, several clinical studies including cancer patients failed to demonstrate objective responses. However, immunostimulatory gene therapy is starting to seem like a viable alternative as new methods for managing cancer patients' continuing immunosuppression emerge [14]. OVs have concurrently been shown to be safe in people. These two disciplines are increasingly combining, and OVs are equipped with immunostimulatory transgenes to boost efficiency and extend immune activation. These new drugs are vying for clearance to become recognized cancer immunotherapies [15,20-22].

It is now known that most viruses can multiply considerably more readily in cancer cells than in normal cells because defence systems against viral infection such as the IFN-β signalling pathway are compromised in most cancer cells [23]. Thus, it is not grim to get a virus to replicate in cancer cells; rather, the challenge is in getting it to completely stop replicating in normal cells while maintaining its capacity to reproduce in cancer cells. In an effort to establish virus replication targeted to cancer cells, efforts have been undertaken to choose a virus that is non-virulent in people or to change the viral genome.

## Conclusions

The use of OVT holds promise for groundbreaking discoveries in the field of cancer research because of their therapeutic effectiveness, fewer side effects, and safety. After the success of immunotherapy with immune checkpoint inhibitors, OVT may represent the next significant advancement in the treatment of cancer. We can safely state that OVT is a well-established cancer treatment modality at this point. The effectiveness of oncolytic virus treatment is anticipated to increase when coupled with immunotherapy options. The primary restriction on oncolytic viruses is the host's quick production of neutralizing antibodies, which reduces the virus's ability to spread to metastatic cancer locations and its potential for therapeutic benefit. Comprehending the dynamic relationship between the immune system and viral oncolysis is crucial for the intelligent design of oncolytic viruses and the formulation of suitable combinatorial approaches. The common characteristic that plays a significant role in showing anticancer effects during oncolytic activities is the formation of specific antitumour immunity with newer potential approaches. Functional transgenes would enable OVs to be equipped with a wide range of anticancer capabilities in the future. OVT seems to be the therapeutic option of choice for cancer patients in a new age of cancer care that is just getting started.
